# Insulin-Dependent H_2_O_2_ Production Is Higher in Muscle Fibers of Mice Fed with a High-Fat Diet

**DOI:** 10.3390/ijms140815740

**Published:** 2013-07-29

**Authors:** Alejandra Espinosa, Cristian Campos, Alexis Díaz-Vegas, José E. Galgani, Nevenka Juretic, César Osorio-Fuentealba, José L. Bucarey, Gladys Tapia, Rodrigo Valenzuela, Ariel Contreras-Ferrat, Paola Llanos, Enrique Jaimovich

**Affiliations:** 1 School of Medical Technology, Faculty of Medicine, University of Chile, Santiago 8380455, Chile; E-Mails: cristian.campos.19@gmail.com (C.C.); adiazvega@gmail.com (A.D.-V.); 2 Center for Molecular Studies of the Cell, Santiago 8380453, Chile; E-Mails: caosoriof@gmail.com (C.O.-F.); acontrerasf@gmail.com (A.C.-F.); pllanosv@gmail.com (P.L.); ejaimovi@med.uchile.cl (E.J.); 3 Nutrition Department, Faculty of Medicine, University of Chile, Santiago 8380453, Chile; E-Mail: jgalgani@med.uchile.cl; 4 Faculty of Medicine, Institute of Biomedical Sciences, Santiago 8380453, Chile; E-Mails: njuretic@med.uchile.cl (N.J.); gtapia@med.uchile.cl (G.T.); rvalenzuelab@med.uchile.cl (R.V.); 5 School of Medicine, University of Valparaíso, Valparaíso 2341369, Chile; E-Mail: jose.bucarey@uv.cl

**Keywords:** obesity, NOX2, insulin resistance, apocynin

## Abstract

Insulin resistance is defined as a reduced ability of insulin to stimulate glucose utilization. C57BL/6 mice fed with a high-fat diet (HFD) are a model of insulin resistance. In skeletal muscle, hydrogen peroxide (H_2_O_2_) produced by NADPH oxidase 2 (NOX2) is involved in signaling pathways triggered by insulin. We evaluated oxidative status in skeletal muscle fibers from insulin-resistant and control mice by determining H_2_O_2_ generation (HyPer probe), reduced-to-oxidized glutathione ratio and NOX2 expression. After eight weeks of HFD, insulin-dependent glucose uptake was impaired in skeletal muscle fibers when compared with control muscle fibers. Insulin-resistant mice showed increased insulin-stimulated H_2_O_2_ release and decreased reduced-to-oxidized glutathione ratio (GSH/GSSG). In addition, p47^phox^ and gp91^phox^ (NOX2 subunits) mRNA levels were also high (~3-fold in HFD mice compared to controls), while protein levels were 6.8- and 1.6-fold higher, respectively. Using apocynin (NOX2 inhibitor) during the HFD feeding period, the oxidative intracellular environment was diminished and skeletal muscle insulin-dependent glucose uptake restored. Our results indicate that insulin-resistant mice have increased H_2_O_2_ release upon insulin stimulation when compared with control animals, which appears to be mediated by an increase in NOX2 expression.

## 1. Introduction

Insulin resistance is a condition present in type 2 diabetes and metabolic syndrome characterized by impaired glucose uptake in target tissues, which produces an imbalance in glucose homeostasis that ultimately may lead to chronic hyperglycemia. Molecular mechanisms involved in the pathophysiology of insulin resistance are related to several alterations in the insulin-signaling cascade [1]. Many molecular defects, such as reduced insulin receptor tyrosine phosphorylation, decreased IRS-1 tyrosine phosphorylation and impaired PI3K activation, have been reported in both skeletal muscle [2] and adipocytes [3]. In the past few years, a series of intracellular molecular alterations related to a highly oxidant intracellular environment have been associated with insulin resistance and obesity [4,5]. Reactive oxygen species (ROS) are involved in many physiological processes. Indeed, H_2_O_2_ is considered a second messenger. However, ROS overproduction and/or insufficient antioxidant mechanisms will alter the cellular redox balance, leading to pathological conditions. One of the best examples of this situation is obesity. Obesity is a major risk factor for insulin resistance, type 2 diabetes and cardiovascular disease. HFD can increase mitochondrial H_2_O_2_ emission potential, a factor contributing to a more oxidized redox environment [1]. Free fatty acids also enhance mitochondrial ROS generation, activate stress kinases and impair skeletal muscle insulin signaling activity. All these effects can be prevented by NAC [6]. It has been proposed that elevated mitochondrial H_2_O_2_ emission is a primary cause for insulin resistance [7]. In addition, HFD also leads to elevated intramuscular triglyceride content, which is also accompanied by increased muscle diacylglycerol and ceramides, both lipid species being activators of protein kinase C [8]. We have previously reported that NOX2 is activated by PKC in skeletal muscle [9]. Considering this evidence, we evaluated the role of NOX2 as a possible contributor to a higher pro-oxidant environment present in obesity and insulin resistance. Molecular modifications triggered by ROS include lipid adducts formation, protein *S*-nitrosylation and protein glutathionylation [5,6]. Particularly, in skeletal muscle of obese mice, an increase in *S*-nitrosylated proteins related to the insulin downstream cascade has been observed and proposed to decrease insulin-signaling activity [5,7]. The increase in intracellular oxidative stress is associated with impaired insulin-dependent glucose uptake. Treatment of L6 muscle cells with 4-hydroxy-2-nonenal disrupted both the insulin signaling pathway and glucose uptake [8]. Oxidant agents, such as H_2_O_2_, trigger the activation of a serine/threonine kinase that phosphorylates multiple targets, including the insulin receptor and IRS proteins. It has been proposed that phosphorylation of the insulin receptor and IRS proteins on serine/threonine residues compete with phosphorylation on tyrosine, the latter being needed for the first events on the insulin cascade [9]. We reported that insulin produces H_2_O_2_ as part of its physiological effects in skeletal myotubes [10], and we showed that insulin-dependent calcium signals in skeletal myotubes are dependent on H_2_O_2_ generated by NOX2 [10]; however, whether an insulin-resistant condition is related with a different pattern of insulin-dependent H_2_O_2_ generation remains unknown.

The aim of this work was to evaluate H_2_O_2_ generation upon insulin stimulation and the possible involvement of NOX2 in the pathophysiology of insulin resistance.

## 2. Results and Discussion

### 2.1. Establishing an Insulin Resistance Model

**Figure 1:**
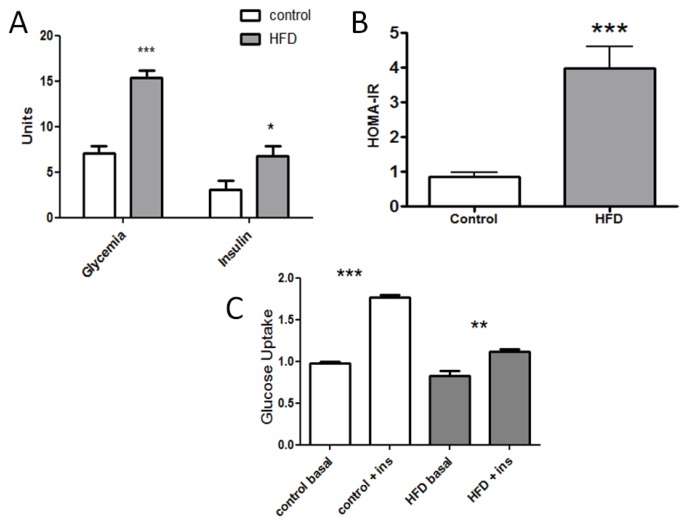
Treatment with a high fat diet during eight weeks induced insulin resistance in mice. (**A**) Glycemia (mmol/L) and insulin (μU/mL) concentration obtained after 14 h fasting (*n* = 17, *t*-Student, * = *p* < 0.02); (**B**) Insulin resistance condition determined by the homeostasis model of assessment-insulin resistance (HOMA-IR) in both control and high fat diet (HFD) mice (*n* = 15, *t*-Student, * = *p* < 0.023); (**C**) Glucose uptake induced by insulin. Cultured skeletal fibers were loaded with *2*-NBDG during 15 min, and then, fluorescence images were acquired. The graph represents relative fluorescence with respect to basal control. Insulin (ins) treated fibers were pre-incubated during 15 min with 100 nM of insulin (*n* = 6, ANOVA, *p < 0.05, **p < 0.01, ***p < 0.005).

In order to obtain a colony of insulin resistant mice, animals were fed with a HFD during eight weeks. Treated animals presented an increased fasting glycemia and serum insulin concentration; glycemia was significantly higher in HFD fed mice compared to control, and insulin concentration was two-fold higher in HFD fed mice than in control (Figure 1A). Consequently, the homeostasis model of assessment-insulin resistance (HOMA-IR) was 0.84 ± 0.14 in the control group and 3.98 ± 0.61 in HFD fed mice (Figure 1B). These results indicate that mice treated with HFD had systemic insulin resistance after eight weeks of feeding. To show that insulin resistance was also present in skeletal muscle, fibers from FDB muscle were stimulated with 100 nM insulin and then incubated with 2-NBDG, to assess glucose incorporation into single fibers from both mice groups. As shown in Figure 1C, mice fed with a standard diet showed a 1.6-fold increased glucose uptake compared to the non-insulin-stimulated condition, whereas animals fed with HFD exhibited a lower increase in glucose uptake upon insulin stimulation (1.1-fold, *p* < 0.05). These results indicate that mice treated with a HFD developed skeletal muscle insulin resistance. Systemic glucose homeostasis is a complex process where liver, adipose tissue and skeletal muscle play a crucial role. Our results show that HFD induce systemic insulin resistance and fasting hyperglycemia. Skeletal muscle insulin resistance can be evidenced by a reduction in insulin-stimulated glucose uptake of both isolated muscle fibers [11] and muscle fiber strips [12]. HFD-induced insulin resistance was evidenced by significantly elevated plasma insulin levels and HOMA-IR compared to control mice, as others have previously reported [13]. However, we show a direct effect of HFD treatment on insulin-dependent glucose uptake in mature, dissociated single skeletal muscle fibers. The methodology using a fluorescent glucose analog allows us to measure glucose incorporation, disregarding the effects of other cell types, like fibroblasts and myoblasts.

### 2.2. H_2_O_2_ Generation Is Higher in Muscle Fibers from High-Fat Diet Mice

**Figure 2:**
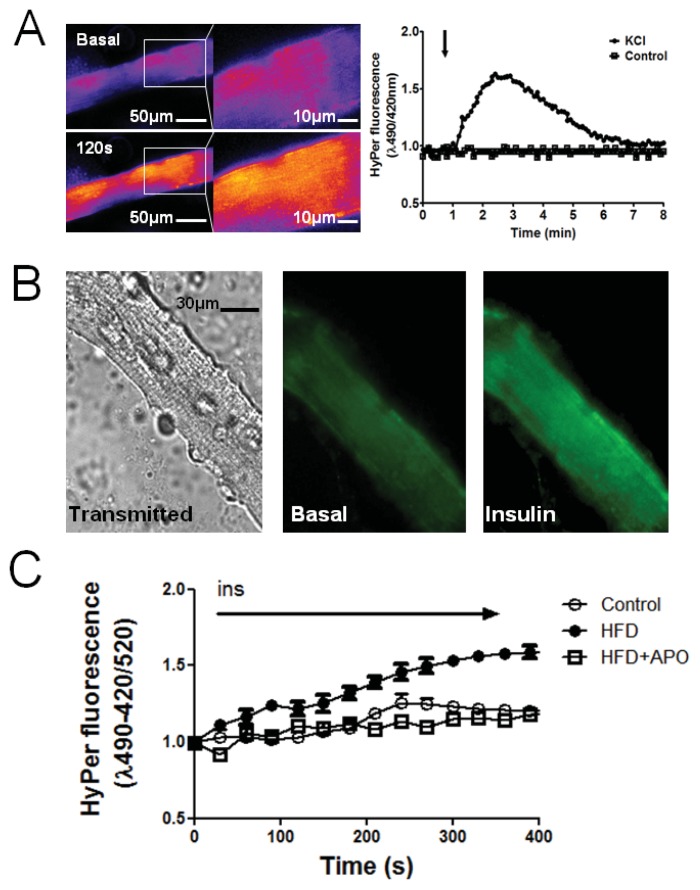
High-fat diet (HFD) effects on H_2_O_2_ production. (**A**) H_2_O_2_ generation was measured before and after 45 mM K^+^ addition. Left panel shows fluorescence in pseudo-color in basal and 120 s after depolarization. Right panel shows the kinetics of depolarization-induced H_2_O_2_; (**B**) Transmitted light and HyPer fluorescence image of a single fiber; (**C**) Time course of changes in the fluorescence ratio of HyPer-Cyto upon addition of 100 nM insulin (→) to muscle fibers of control and high-fat diet mice (HFD) and mice pre-incubated with apocynin (15 min) (50 μM APO) (mean ± SEM). Radiometric changes are shown; images were acquired using an excitation/emission wavelength λ_exc1_−λ_exc2_/λ_em_ = 420−490/520 nm. We normalized the ratio of basal fluorescence in muscles from animals under different conditions.

Fibers from flexor digitorum brevis (FDB) muscle were transfected with the genetically encoded fluorescence sensor HyPer plasmid to evaluate whether insulin is capable of inducing H_2_O_2_ generation, as has been previously described in cultured myotubes [10]. We successfully expressed the HyPer protein in the cytosol (HyPer-Cyto) of mature skeletal fibers. We have reported that membrane depolarization produces an increase in ROS, measured using a (5-(and-6)-chloromethyl-2′, 7′-dichlorodihydrofluorescein diacetate probe [14]; we now tested HyPer-Cyto response after depolarization. Fibers were stimulated with a 47 mM K^+^ solution, and the change in fluorescence ratio was recorded (Figure 2A). Depolarization produced a transient increase in ROS generation in fibers that were previously incubated with N-benzyl-p-toluenesulfonamide (BTS), to abolish an effect due to contraction.

Figure 2B shows a transmitted image from a single adult fiber and the fluorescence of a transfected cell before and after 120 s stimulation. In skeletal fibers, 100 nM insulin triggered a slight H_2_O_2_ increase after stimulus; a change of 20% in the fluorescence ratio over basal ratio, 30 s after stimulation, was detected, and the ratio remained constant during 5 min after stimulation (Figure 2C). In HFD fibers, insulin-dependent fluorescence of HyPer-Cyto reached a peak 50% higher than basal, 150 s after stimulus (Figure 2B,C). These results point to a higher production of H_2_O_2_ by skeletal muscle from insulin-resistant mice in response to insulin. A main source of H_2_O_2_ induced by insulin is NOX2, and apocynin is a classical NOX2 assembly inhibitor and, as such, impairs NOX2 activation. H_2_O_2_ kinetics generated by insulin was similar in HFD-fed mice pre-incubated with apocynin compared with control mice. This result points to a direct role of NOX2 elevating the H_2_O_2_ levels in skeletal muscle of insulin resistance mice. HyPer is a H_2_O_2_-selective molecular probe that has advantages in terms of specificity and reversibility over non-specific fluorescent probes for ROS measurement, such as (5-(and-6)-chloromethyl-2′,7′-dichlorodihydrofluorescein diacetate. Mature muscle fibers can be transfected using an *in vivo* electroporation protocol [15], but here, we show a variant that allows us to work on mature fibers with a very simple transfection protocol, avoiding an invasive procedure on the animal. Our results indicate that skeletal muscle from insulin resistance mice generates higher insulin-dependent H_2_O_2_ levels. Skeletal muscle expresses two isoforms of NADPH oxidase, NOX2 and NOX4 [16]; only NOX2 needs the p47^phox^-dependent assembly of the complex at the plasma membrane to form the membrane-associated flavocytochrome b588 protein [17]. Besides NOX2, H_2_O_2_ is also generated by xanthine oxidase and during oxidative phosphorylation in mitochondria [18]. The fact that muscle glutathione oxidation is prevented by apocynin suggests that NOX2 is one of the sources of H_2_O_2_. However, we cannot exclude that apocynin may have a non-specific antioxidant role, which may also decrease ROS generation from other sources, including mitochondria. In agreement with our results, Yokota *et al*. showed that NADPH oxidase activity was increased in skeletal muscle of HFD fed mice and was inhibited by apocynin treatment [19].

It is worth noting that fibers from HFD animals do not increase glucose transport to the same level of controls in response to insulin, but they did produce H_2_O_2_ in response to the same concentrations of insulin. This means that NOX2 activation by insulin occurs through a pathway other than the metabolic signal. If insulin resistance is due to decreased traditional signaling through the insulin receptor, presumably the increased hydrogen peroxide is due to higher expression of NOX2. On the other hand, it has been shown that H_2_O_2_ production may negatively affect the insulin signaling pathway through dephosphorylation of the insulin receptor and its tyrosine-phosphorylated substrates, as well as by increasing serine phosphorylation of the insulin receptor and IRS-1 [20,21]. Evidence in the literature highlights a possibly relevant role of ROS in triggering both insulin resistance and type 2 diabetes [13,22,23]. Here, we show direct evidence that those animals with insulin resistance produce higher amounts of H_2_O_2_ in the presence of the same doses of insulin compared to control animals. The fact that apocynin, at doses reported to inhibit NOX2 activity, is capable of not only restoring plasma glucose levels, but also of reducing plasma insulin levels in insulin resistance mice, preventing intracellular oxidative increase, suggests that this drug or its derivatives, such as vanillin [24], should be considered in future studies as a therapy for insulin resistance.

### 2.3. Skeletal Muscle GSH Content in Insulin-Resistant Mice

**Figure 3:**
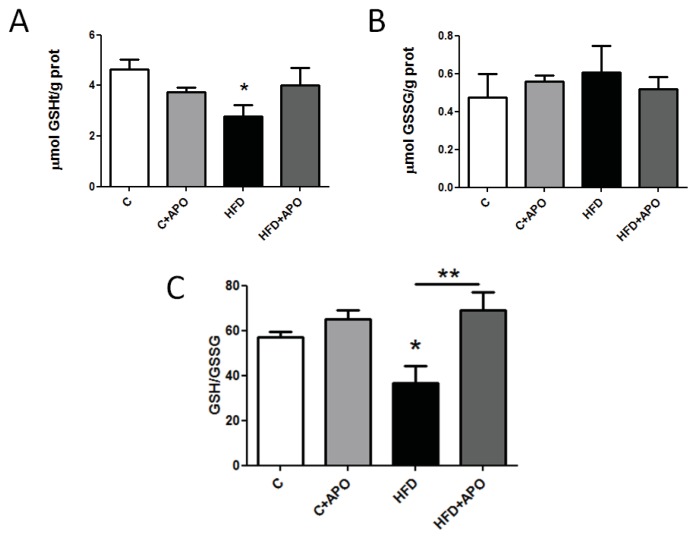
Apocynin effects on glutathione concentration. Control and insulin resistance mice were used after 14 h fasting. Total (tGSH) (**A**) and oxidized (GSSG) (**B**) glutathione concentrations were determined in tibialis anterior (TA) skeletal muscles through an enzymatic recycling method (Oxis Research). GSH/GSSG ratio is shown (**C**). All measurements were normalized to protein content (g). APO: mice treated with apocynin during eight weeks (*n* = 6, ANOVA, Newman-Keuls, *p < 0.06). GSSG (*n* = 6, ANOVA, Newman-Keuls, *p < 0.05).

To test for a possible higher oxidative intracellular environment in HFD mice due to chronic H_2_O_2_ production, we measured the amount of reduced (GSH) and oxidized (GSSG) glutathione in tibialis anterior (TA) muscle from HFD fed mice. The amount of total GSH was higher in control animals compared with muscle of HFD fed mice (Figure 3A). In contrast, apocynin treatment did not affect GSH content in neither control nor insulin resistance mice. In addition, HFD did not substantially change muscle GSSG content when compared with chow diet fed mice (Figure 3B). Apocynin decreased GSSG levels of control mice, but the apparent decrease in GSSG in HFD-treated mice was not statistically significant. The ratio of GSH/GSSG obtained in the HFD-treated group was lower than that in the control group. The significant reduction in the GSH/GSSG ratio induced by HFD (Figure 3C) was prevented in HFD mice treated with apocynin (Figure 3C). These results show a chronic pro-oxidant intracellular environment in insulin-resistant animals, which can be prevented by the administration of apocynin. It is important to note that the increased pro-oxidant status in skeletal muscle was accompanied by impaired glucose tolerance. Overexpression of NOX2 subunits was described in vascular endothelial tissue from obese patients; it was also accompanied by increased oxidative stress and upregulation of antioxidant enzymes [25]. In a different cellular model (pancreatic islets), it has been shown that free-fatty acids increase superoxide production through NADPH oxidase activation [26,27].

### 2.4. Skeletal Muscle NOX2 Expression in Insulin-Resistant Mice

**Figure 4:**
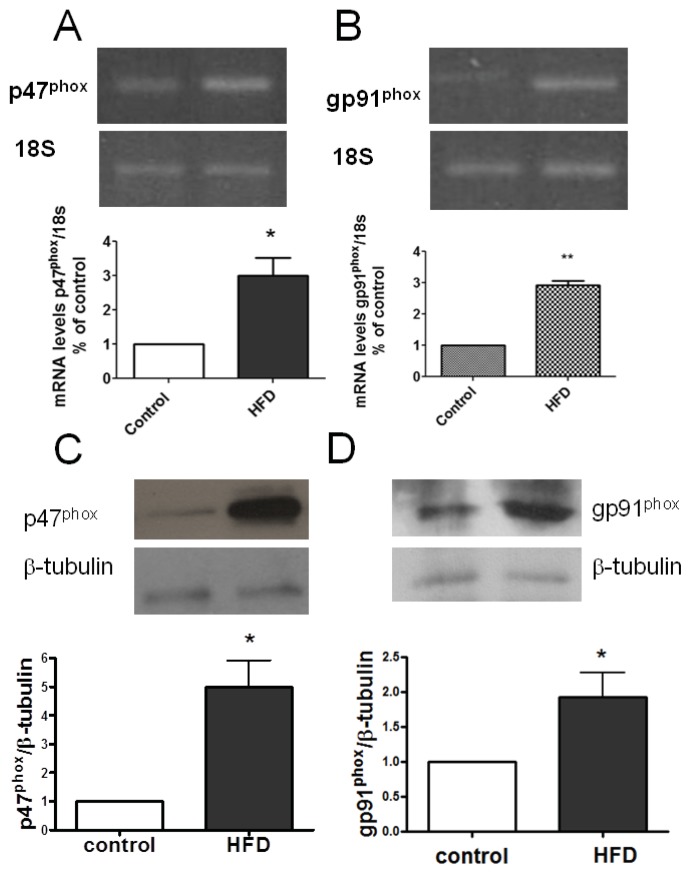
HFD treatment produces increased levels of both p47^phox^ and gp91^phox^ mRNA and protein in skeletal muscle. Control and insulin resistance mice were used after 14 h fasting. After euthanasia, tibialis anteriors (TAs) were dissected and triturated in TRIzol reagent. mRNA levels were analyzed by semiquantitative RT-PCR. Characteristic agarose gels of RT-PCR products are shown in the upper panel, (**A**) and (**B**). Results were normalized to 18S expression (mean ± SEM, *n* = 3). *p < 0.05; **p < 0.02; (**C**) Western blot and densitometry analysis from TA (control or HFD mice); incubations with primary antibody were overnight at 4 °C with primary antibodies: anti-p47^phox^, 1:1000, *n* = 3; (**D**) Western blot and densitometry analysis from TA of gp91^phox^ (membrane subunit of NOX2). Results were normalized to the β-tubulin protein level and presented as a fold over untreated control cells (mean ± SEM; *n* = 3, *p < 0.05 *t*-Student test was applied).

Considering that muscle fibers from insulin-resistant mice display a higher H_2_O_2_ generation after insulin addition, we evaluated whether skeletal muscle (tibialis anterior) mRNA and protein levels for p47^phox^ and gp91^phox^ (subunits of NOX2) are over-expressed in skeletal muscle from these mice. HFD fed mice had about a 3-fold increase in p47^phox^ and gp91^phox^ over the control (Figure 4A,B). Western blot analysis showed that p47^phox^ protein levels were near 7-fold over control in TA muscle from insulin-resistant mice; and, in turn, gp91^phox^ was 1.6-fold over control (Figure 4C,D). Both results indicate that insulin-resistant mice have a higher expression of NOX2 in skeletal muscle.

### 2.5. Apocynin in the Diet Prevents HFD-Induced Insulin Resistance in Mice

**Figure 5:**
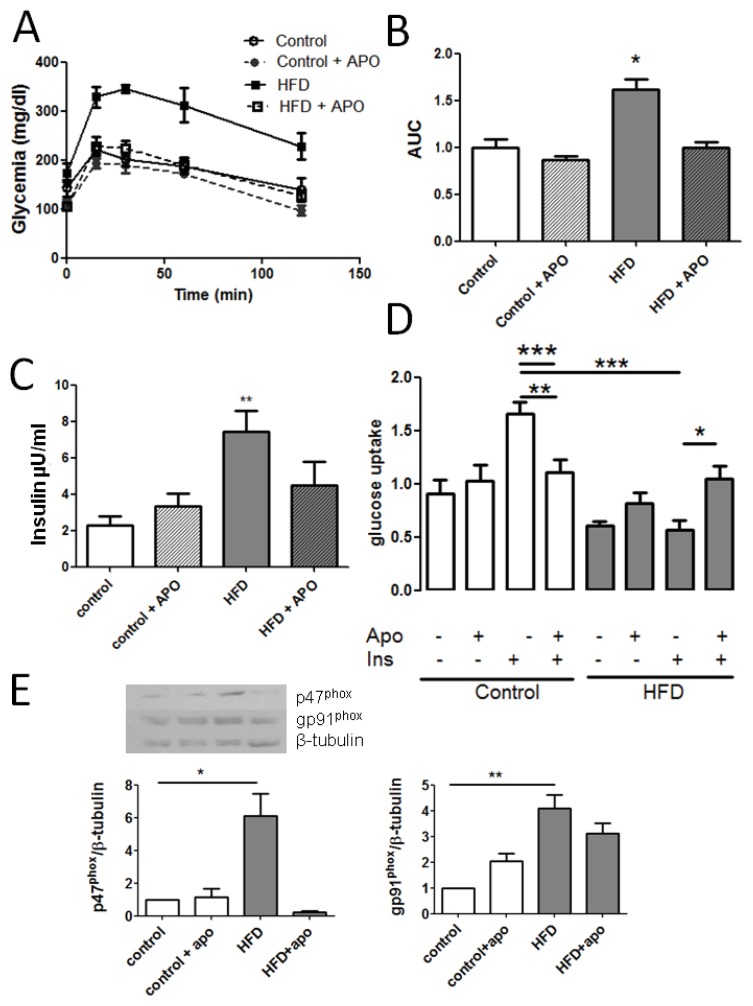
Apocynin prevents insulin resistance in mice. (**A**) Oral glucose tolerance curve performed for 14 h fasting. Glucose intake was 2 g/kg weigh (*n* = 6 for each condition); (**B**) Area under curve of (**A**); (**C**) Fasting insulin concentration in control, HFD (high-fat diet fed animals) and APO (apocynin treated mice, *n* = 6); (**D**) Glucose uptake in basal and insulin-stimulated condition from isolated cultured fibers pre-incubated during 2 h with 500 μM apocynin (APO) (8–12 fibers, *n* = 4, for each condition); (**E**) Western blot against p47^phox^ and gp91^phox^ from TA; mice were treated or not with apocynin during eight weeks (*n* = 3, ANOVA, Newman-Keuls. ***p < 0.001, **p < 0.005, *p < 0.01).

Apocynin treatment of mice during the eight week period of differential feeding was aimed to maintain a constant inhibition of NOX2. We used a dose reported by others [28]. An oral glucose tolerance test (OGTT) was performed after 14 h fasting, to control the impairment in glucose tolerance. HFD-fed mice had impaired glucose control in fasting, as well as after glucose stimulation (Figure 5A,B). Apocynin treatment did not affect glucose tolerance when it was provided with control diet. However, when it was provided in combination with HFD diet, it prevented the impairment of glucose tolerance associated with this diet. Apocynin also produced a significant decrease in insulin levels observed in HFD fed mice (7.4 ± 1.13 in HFD and 4.5 ± 1.30 μU/mL in HFD + apocynin, Figure 5C). Apocynin blocks the interaction between p47^phox^ and gp91^phox^, and NOX4 does not require p47^phox^ for its activation [17].

We measured glucose uptake in the presence of apocynin, and we found that apocynin inhibited glucose uptake in control fibers, which is consistent with the proposed role of H_2_O_2_ as mediator in the insulin pathway [10,21]. In HFD skeletal fibers, the acute treatment with apocynin produced an effect opposite to that observed in control; apocynin increased the low levels of glucose uptake present in HFD-fibers. We can speculate that apocynin produced this effect by lowering the chronic excess of H_2_O_2_ characteristic of the insulin resistance condition, associated to a higher serine phosphorylation. On the other hand, apocynin administrated together with the HFD prevents the increase in p47^phox^ protein levels, contributing both to lower the level of ROS production and to improve the systemic insulin sensibility.

Apocynin has been used already to inhibit NADPH oxidase in skeletal muscle [29], and the dose used here has been administered to C57BL/6J and KKAy mice, showing that apocynin reduced oxidative stress in fat tissue [28]. Apocynin is an inhibitor of NOX2, and NOX2 is strongly expressed in phagocytic cells. Obesity is a state characterized by macrophage infiltration of the liver [30] and adipose tissue [31]. It has been proposed that such an inflammatory state leads to increased ROS production and impaired insulin sensitivity. Apocynin could lead to inhibition of NOX2 present in macrophage cells and not necessarily of the enzyme expressed in muscle cells. Moreover, apocynin has also been shown to induce the expression of hepatic antioxidant enzymes, which may also contribute to ameliorate HFD-induced insulin resistance [32]. There is evidence that points to an antioxidant role of apocynin independent of NADPH oxidase inhibition in vascular tissue [33]; although we have no evidence to rule out a possible antioxidant role of apocynin, since we have previously reported that siRNA against p47^phox^ is able to inhibit the insulin-dependent H_2_O_2_ production [10], the most probable explanation of the effect of the drug in skeletal muscle shown in this work is through the inhibition of NOX2, as previously described. HFD has been described to promote a pro-oxidant environment through an elevation in mitochondrial H_2_O_2_-emitting potential and a reduction in the GSSG/GSH ratio. Such features have proven to be prevented by the administration of an antioxidant peptide localized in the inner mitochondrial membrane [34]. We do not discard the participation of mitochondria [35] in the insulin-dependent H_2_O_2_ signal observed in our study.

## 3. Experimental Section

### 3.1. Animals

Male C57BL/6J mice were obtained from the Animal Facility at the Faculty of Medicine, University of Chile. Room temperature was kept constant at 21 °C, and light was maintained on a 12:12 h light-dark cycle. At 20 days of age, mice were divided into four diet groups. The control group was treated with a diet containing (*wt*/*wt*) 10% fat, 20% protein and 70% carbohydrate. The high-fat diet (HFD) group received a diet containing (*wt*/*wt*) 60% fat, 20% protein and 20% carbohydrate (D12492, Research diets, New Brunswick, NJ, USA). Two groups (one control and one HFD fed) of animals were treated with 5 mM apocynin added to the drinking water during 8 weeks, as previously described [28], simultaneously with the respective diets. Animals were sacrificed after 8 weeks. All the procedures performed in this work were approved by the Bioethics Committee of the Faculty of Medicine, University of Chile.

### 3.2. Biochemical Determinations

An oral glucose tolerance test (OGTT) was performed after 12–14 h fasting by administration of a glucose bolus of 2 g/kg via gavage through a gastric tube. At 0, 15, 30, 60 and 120 min, tail blood samples were obtained. Blood glucose concentrations were measured on a Johnson and Johnson OneTouch Glucometer. Plasma insulin concentrations were determined by a commercially available immunoassay specific for mice (Mercodia, Uppsala, Sweden).

### 3.3. Single-Cell Fluorescent 2-NBDG Uptake Assay

Muscle fibers were washed with Krebs buffer (in mM: 20 HEPES-Tris, pH 7.4, 118 NaCl, 4.7 KCl, 3 CaCl_2_, 1.2 MgCl_2_ and 10 glucose) and stimulated with 100 nM insulin for 15 min. Cells were exposed to 2-[*N*-(7-nitrobenz-2-oxa-1,3-diazol-4-yl)amino]-2-deoxy-D-glucose (2-NBDG, 300 μM) for 15 min, rinsed with Krebs buffer before stimulus. Cultures were excited at 488 nm, and the fluorescence was captured by 505–550 nm band pass filter emission. 2-NBDG uptake was estimated by comparing intracellular fluorescence with the signal from outside the cells. Pascal 5 microscope and PlanApofluo 40X (numerical aperture 1.3) (LSM 5 PASCAL, Carl Zeiss, Thornwood, NY, USA) was used. Image J software (NIH, Bethesda, MD, USA) was used to quantify 2-NBDG uptake.

### 3.4. Fibers Transfection and H_2_O_2_ Measurement

We used a plasmid that encodes for HyPer protein to measure H_2_O_2_ production. Plasmid was acquired from Evrogen Joint Stock Company (Moscow, Russia). Fibers were transfected using Lipofectamin 2000 (Invitrogen, Carlsbad, CA, USA) for 2 h (1 μg DNA/3 μL during collagenase digestion of flexor digitorum longus (FDB) muscle. H_2_O_2_ generation was determined in skeletal muscle fibers 24 h after transfection. Images were acquired using an Olympus IX81-DSU Spinning Disk Confocal Microscope. HyPer fluorescence was detected using an excitation/emission wavelength λ_exc1_−λ_exc2_/λ_em_ = 420−490/520 nm. The ratio between the signals excited with 490 and 420 nm was used to determine the presence of H_2_O_2_, HyPer has a 420 nm excitation peak that decreases in proportion to the increase at 490 nm. Fluorescence emitted at 520 nm was shown. Each experiment was performed alongside the effect of laser excitation alone. Noise in the images was removed using Image J Filters [36].

### 3.5. Glutathione (GSH) Measurement

GSH concentration was measured using a glutathione assay kit (OxisReseach, Portland, OR, USA). Briefly, tibialis anterior (TA) was dissected and then crushed using Tissue Tearor (BioSpec Products, Bartlesville, OK, USA) in PBS plus 5% metaphosphoric acid, 0.6% sulfosalicylic acid and 0.01% triton X-100. The mix was divided in two samples; one of them was treated with 1-methyl-2-vinyl-pyridinium trifluoromethane, to measure oxidized glutathione (GSSG), and the other one was used to measure GSH. Samples were centrifuged at 3000× *g* by 10 min at 4 °C; the supernatant was used for measurements. Proteins were measured to normalize the results and were determined by Coomassie Plus (Bradford) Protein Assay (Thermo Scientific, Rockford, IL, USA).

### 3.6. Western Blot Analysis

Tibialis anterior (TA) muscles from mice were homogenized in cold lysis buffer (140 mM NaCl; 0.1% triton X-100 and 1 mM TRIS, pH 7.4) using Tissue Tearor. Samples were incubated on ice for 1 h. after centrifugation for 30 min to 3000× *g*, supernatant proteins were separated on 10% SDS-PAGE gel. After transference to polyvinylidene difluoride membrane, incubations with primary antibody were maintained at 4 °C overnight with the primary antibodies: anti-p47^phox^, 1:800 (Santa Cruz Biotechnology, Dallas, TX, USA), gp91^phox^ 1:1000 (BD Biosciences, San Jose, CA, USA) and anti-β-tubulin 1:4000 (Sigma-Aldrich, St. Louis, MO, USA). Secondary antibodies, anti-rabbit and anti-mouse (Sigma-Aldrich, St. Louis, MO, USA) were incubated during 1.5 h.

### 3.7. RT-PCR

Total RNA from skeletal fibers were extracted using TRIzol Reagent (Invitrogen, Carlsbad, CA, USA), and cDNA was prepared by using SuperScrip II, RNAse H-RT (Invitrogen). cDNA was amplified using mouse-specific gp91^phox^ and p47^phox^ primers [37]. mRNA concentration was normalized to 18S expression.

The primers used were: gp91^phox^: 5′- TCACATCCTCTACCAAAACC-3′ (sense) and 5′- CCTTTATTTTTCCCCATTCT-3′ (antisense). p47^phox^: 5′- AGAACAGAGTCATCCCACAC-3′ (sense) and 5′- GCTACGTTATTCTTGCCATC-3′ (antisense). 18S: 5′- AGTTGGTGGAGCGATTTGTC-3′ (sense) and 5′- TATTGCTCAATCTCGGGTGG-3′ (antisense). PCR amplification was maintained in the exponential phase for each product. PCR conditions were: one cycle of 95 °C for 2 min, followed by 37 cycles at 95 °C for 30 s, *X* °C for 30 s, 72 °C for 30 s and a final cycle of 10 min at 72 °C (*X* = 53 °C for gp91^phox^ and 55 °C for p47^phox^ and 18 S).

PCR products were resolved by electrophoresis on 2% agarose gel and stained with ethidium bromide (gp91^phox^: 198 bp; p47^phox^: 247 bp and 18S: 143 bp). Bands were quantified by densitometric analysis using the Scion Image program from NIH.

### 3.8. Statistics

Data are presented as the mean ± SEM. Significant differences between and within multiple groups were examined using ANOVA for repeated measures, followed by Newman-Keuls multiple comparison test. The Student *t*-test was used to detect significant differences between two groups. *p* < 0.05 was considered statistically significant.

## 4. Conclusions

We demonstrated that skeletal muscle from HFD fed animals has a pro-oxidant environment accompanied by increased expression of NOX2 subunits; this appears to be an important factor to generate H_2_O_2_ in response to insulin. This is the first report to show direct evidence that insulin resistance is characterized by a higher insulin-stimulated H_2_O_2_ generation in skeletal muscle, and NOX2 appears to play an essential role in this mechanism. This evidence points to a relevant role of H_2_O_2_ generation in the pathophysiology of insulin resistance.
